# Information Technology in Critical Care: Review of Monitoring and Data Acquisition Systems for Patient Care and Research

**DOI:** 10.1155/2015/727694

**Published:** 2015-02-04

**Authors:** Michael A. De Georgia, Farhad Kaffashi, Frank J. Jacono, Kenneth A. Loparo

**Affiliations:** ^1^University Hospitals Case Medical Center, 11100 Euclid Avenue, Cleveland, OH 44106-5040, USA; ^2^Case Western Reserve University, 10900 Euclid Avenue, Cleveland, OH 44106-7078, USA; ^3^Louis Stokes Cleveland VA Medical Center, 10701 East Boulevard, Cleveland, OH 44106, USA

## Abstract

There is a broad consensus that 21st century health care will require intensive use of information technology to acquire and analyze data and then manage and disseminate information extracted from the data. No area is more data intensive than the intensive care unit. While there have been major improvements in intensive care monitoring, the medical industry, for the most part, has not incorporated many of the advances in computer science, biomedical engineering, signal processing, and mathematics that many other industries have embraced. Acquiring, synchronizing, integrating, and analyzing patient data remain frustratingly difficult because of incompatibilities among monitoring equipment, proprietary limitations from industry, and the absence of standard data formatting. In this paper, we will review the history of computers in the intensive care unit along with commonly used monitoring and data acquisition systems, both those commercially available and those being developed for research purposes.

## 1. Introduction

There is a broad consensus that health care in the 21st century will require the intensive use of information technology and clinical informatics to acquire and manage data, transform the data to actionable information, and then disseminate this information so that it can be effectively used to improve patient care. Nowhere is this more evident and more important to patient outcomes than in the intensive care unit (ICU). Critical care involves highly complex decision making. It is by nature data-intense. Despite the growth of critical care, however, the basic approach of data collection and management has remained largely unchanged over the past 40 years. Large volumes of data are collected from disparate sources and reviewed usually retrospectively; and even that is difficult. Providers must navigate through a jungle of monitors, screens, software applications, and often paper charts that provide supplemental patient data inherent in today's cacophony of information management systems. Data from patient monitors and medical devices, although available visually at the bedside, is challenging to acquire and store in digital format. There is limited medical device interoperability and integration with the electronic medical record (EMR) remains incomplete at best and cumbersome.

In addition (and partly as a result of these limitations), standard analytical approaches provide little insight into a patient's actual pathophysiologic state. Understanding the dynamics of critical illness requires precisely time-stamped physiologic data (sampled frequently enough to accurately recreate the detail of physiologic waveforms) integrated with clinical context and processed with a wide array of linear and nonlinear analytical tools. This is well beyond the capability of typical commercial monitoring systems. Such an understanding derived from advanced data analytics can aid physicians in making timely and informed decisions and improving patient outcomes. Ultimately, an integrated critical care informatics architecture will be required that includes acquisition, synchronization, integration, and storage of all relevant patient data into a single, searchable database (numeric and waveforms) and data processing to extract clinically relevant features from raw data and translate them into actionable information [1]. Advances in technology are beginning to bring all of this together.

In this paper, we will review the history of computers in the ICU along with commonly used monitoring and data acquisition systems, both those commercially available and those being developed for research purposes.

## 2. Computers in the ICU

Clinical information management systems are now common in most hospitals. These systems have evolved along several parallel lines beginning, not surprisingly, in 1946 with the introduction of the Electronic Numerical Integrator and Computer (ENIAC), the first general-purpose computer (see Table 1). The size of a room and weighing in at 27 tons, ENIAC was developed to calculate missile trajectories for the U.S. Army [2]. Five years later, IBM introduced the first commercially available computer, the Engineering Research Associates (ERA) 1103. Because of their exorbitant cost, use of early computers was limited to large corporations to help manage accounting. In the 1960s, academic institutions followed suit and began developing computer systems to streamline their growing business operations. A decade later, hospitals began to develop EMR systems including the Problem Oriented Medical Record (POMR) at the University of Vermont [3], Health Evaluation through Logical Processing (HELP) at the University of Utah [4], and The Medical Record (TMR) at Duke University [5] and the Computer Stored Ambulatory Record (COSTAR) at Harvard [6]. COSTAR was programmed in MUPMS (Massachusetts General Hospital Utility Multi-Programming System), a computer language better formatted for medical data than COBOL and FORTAN, which were routinely used at the time (MUMPS was eventually adopted for use by the Department of Veterans Affairs). While Indiana's Regenstrief Medical Record System (RMRS) was one of the first systems for both in-patient and outpatient settings [7], these early EMRs were rarely connected to the real-time data-intense environment of the ICU. This was a world unto itself.

Shubin and Weil are credited with introducing the computer to the ICU in 1966 for the purpose of automatically collecting vital signs from the bedside monitor [8]. By connecting an IBM 1710 computer through an analog-to-digital converter to bedside devices, they were able to collect arterial and venous pressure, heart rate, temperature, and urinary output. This had actually been done before in the operating room, though not easily. Using a mechanical contraption, McKesson recorded tidal volumes, fraction of inspired oxygen, and blood pressure in 1934 [9]. The development of the computer (and particularly the microprocessor) made this onerous task much easier. Basic analytical tools, such as trend analysis, were soon added to the automated data collection systems [10]. Other early applications of computers in medicine included one of the first clinical decision support systems to aid in the diagnosis of hematologic disorders by Lipkin and colleagues [11], systems for respiratory monitoring by Stacy and Peters [12], and automation of blood transfusion after cardiac surgery by Sheppard and colleagues [13]. The computer-based Clinical Assessment, Research, and Education System (CARE) was a clinical decision support system developed to aid in the treatment of critically ill surgical patients. The system continuously monitored physiologic and metabolic functions of critically ill patients and managed data about fluid and electrolytes as well as cardiac and respiratory functions [14].

This experience with computers in academic institutions inspired Hewlett-Packard to offer a commercial version of these systems. Adoption of their Patient Data Management System (PDMS), however, was slow because the primitive user interfaces and complex menus were not suited to the fast pace of the ICU [15, 16]. In the 1980s, automatic collection of heart rate and blood pressure became more advanced with data being presented in graphical displays that mimicked the familiar bedside flow sheet [17]. The architecture also evolved from the locally contained model to the client/server model in which a workstation in the ICU (the client) interacted with a central computer housing patient data (the server) via a local area network (LAN). Navigational tools became more user friendly though analytical capabilities remained limited [18]. Links to the fledgling hospital EMR systems were also being made beginning with the computer system that handled admissions, discharges, and transfers (ADT) so that patient demographic data could be readily accessed. Physician and nursing notes were soon being entered electronically into a problem-oriented medical record [19]. In parallel to the ICU, computers were also being introduced in the 1980s into the operating room. Picking up where McKesson left off, in 1986 Gravenstein introduced computerized anesthesia records [20], which allowed for more reliable collection, storage, and presentation of data during the perioperative period as well as provide basic record keeping functions (thus in their infancy such systems were called “anesthesia record keepers”). Still, as in the ICU, data from medical devices were rarely integrated with the other physiological data.

In the 1990s, ICU systems improved significantly with increased clinical functionality and Internet access. Web-based software used Web browsers to display the user interface and simple queries of cumulative patient data were supported. Vendors migrated the technology that had been developed for the OR and the ability to record and present continuous patient data as well as provide links to physician notes, nursing documentation, and laboratory and imaging data from the evolving EMR systems, thus creating large enterprise systems, now broadly referred to as Clinical Information Systems [21].

## 3. Clinical Information Systems

Several Clinical Information Systems are commercially available today for the ICU and competition among vendors is intensifying. Frost & Sullivan have estimated that the annual US market for emergency, perioperative, and intensive care software solutions is currently approximately $842.2 million and are expected to reach $1.3 billion in 2015 [22]. Not one company has a dominant share of the market and several have evolved over the last decade, through various acquisitions of smaller participants, to offer broad end-to-end platforms [23]. For example, GE's Centricity Critical Care system from GE HealthCare (Chalfont St. Giles, UK), introduced in 2003, is the culmination of the acquisitions of, among others, Marquette Medical Systems, a leading manufacturer of patient monitors, Instrumentarium, a manufacturer of mechanical ventilators and anesthesia equipment, and iPath, the basis of the Operating Room Management Information System. For the EMR side, GE also acquired MedicaLogic, a leading provider of outpatient digital health records, and IDX Systems, primarily a practice management and billing system. IDX was written using MUMPS, which currently also forms the basis for EpicCare (Epic Systems Corporation, Verona, WI) and Veterans Health Information Systems and Technology Architecture (VistA). GE's Centricity Critical Care automatically collects data from monitors and ventilators displays it in spreadsheets reminiscent of the typical paper ICU chart. Data are collected from medical devices through device interfaces that connect with GE's Unity Interface Device (ID) network.

Philips Healthcare (Andover, MA) also has a long history in the ICU with the introduction of the Patient Data Management System in the early 1970s under the Hewlett-Packard brand. In the 1990s this became CareVue [24] and the most recent iteration is the IntelliVue Clinical Information Portfolio (ICIP) Critical Care introduced in 2007. Like GE's Centricity, Philips' ICIP Critical Care also evolved through a series of acquisitions [25]. These included Agilent Technologies Healthcare Solutions Group, a leader in patient monitoring and critical care information management, Witt Biomedical Corporation, a leader in hemodynamic monitoring in catheterization laboratories, and Emergin, a developer of alarm management software. In 2008, Philips acquired TOMCAT Systems Ltd., a company that offers software for the collection of cardiac data and that same year, acquired VISICU Inc., a provider of tele-ICU technology. On the EMR side, Philips also partnered in 2004 with Epic in order to provide end-to-end integration with electronic medical records. As with GE's Centricity, Philips' ICIP Critical Care supports automatic (or manual) documentation of physiologic data with time resolutions up to every 5 minutes. Philips Information Support Mart interfaces with ICIP and provides a relational database that archives clinical information such as lab results, text notes, medications, and patient demographics that can be queried with special scripts (see MIMIC II below).

## 4. Limitations of Commercially Available Clinical Information Systems

While modern Clinical Information Systems do provide end-to-end platforms for the ICU, there are several limitations that remain. First, they remain limited in terms of functionality and the acquisition of high-resolution physiologic data. For example, the actual physiological waveform signals are not acquired or stored by either the GE's Centricity system or the Philips ICIP system. This is an important limitation of most commercially available enterprise systems today and is the result of a tradeoff between the memory requirements of capturing* high-resolution physiological data* (including the underlying waveform morphology) versus capturing only data snapshots that may be sufficient for certain clinical decision-making. Currently, no standards have been defined as to where that balance lies. Philips has developed its own proprietary solution for automated acquisition of waveform data for research called the Research Data Exporter (RDE). This solution does not acquire the data at the native sampling rate of the signal and limits the number of waveforms that can be exported. Better real time acquisition of physiological waveform signals is needed along with education of clinicians regarding its value in understanding of complex physiology.

Second, there is currently neither processing nor analysis of data. While a few monitors can display raw trends, even basic analyses (mean, median, standard deviations) are difficult to perform at all let alone in real time and higher-level analyses are impossible. New physiological models are now emerging suggesting that nonlinear changes in dynamics over time may have more predictive value. Understanding this complex physiology can lead to more timely intervention and better outcomes. Techniques for the analysis of nonlinear systems have emerged from the mathematical and engineering sciences but have not been applied to physiological data in the ICU (in part because the acquisition and integration challenges have not been met). The promise of critical care informatics lies in the potential to use these advanced analytical techniques on high-resolution multimodal physiological data in order to have a better understanding of the complex relationships between physiological parameters, improve the ability to predict future events, and thereby provide targets for individualized treatment in real time. In the future, we will use a system that does not simply report streams of raw data to physicians but also synthesizes it to form hypotheses that best explain the observed data, a system that translates multidimensional data into actionable information and provides* situational awareness* to the clinician [26].

Third, visual displays in the ICU have advanced little since bedside electronic monitors were introduced more than four decades ago despite the increasing volume of data collected. For example, clinicians may be confronted with more than 200 variables [27] when caring for critically ill patients yet most people cannot judge the degree of relatedness between more than two [28]. This greatly contributes to preventable medical errors [28]. Graphical displays must be carefully and thoughtfully designed by applying a human systems integration approach. It is important to understand not only how information should be optimally presented to promote a better understanding of the patient's pathophysiologic state and support decision-making but also to facilitate collaboration and work-flow among the team [29]. Finally, despite the recent growth of tele-ICUs, networks of audio-visual communication and computer systems that link ICUs to intensivists, most of these same technical limitations remain. That is, whether these systems are used to monitor a patient located 300 miles away or 3 feet away, the underlying principles, equipment interoperability, data acquisition, synchronization, and data analysis, are equally applicable. Investment in this basic information technology architecture is needed for the next generation of tele-ICU care.

## 5. Medical Device Interoperability and Data Integration

Central to the growth of critical care has been the proliferation of monitoring technology and stand-alone medical devices. For example, a typical critically ill patient may undergo frequent or continuous monitoring of dozens of physiological parameters. An enormous amount of data is generated reflecting dynamic and complex physiology, dynamics that can only be understood by data integration and clinical context. Most of these parameters, however, are generated from stand-alone devices that do not easily integrate with one another. Some connect directly into the bedside monitor but many others do not (or do so incompletely meaning that not all the data is captured electronically). A lack of functional medical device interoperability is one of the most significant limitations in health care today. For example, more than 90% of hospitals recently surveyed by HIMSS use six or more types of medical devices and only about a third integrate them with one another or with their EMRs [30].

In contrast to the “plug and play” world of consumer electronics, most acute care medical devices are not designed to interoperate. Most devices have data output ports (analog, serial, USB, and Ethernet) for data acquisition but there is no universally adopted standard that facilitates multimodal data acquisition and synchronization in a clinical setting; each device often has a unique communication protocol for data transfer and often the time base for each device is independently set rather than determined from a standard source. The development and adoption of medical device standards to improve interoperability is ongoing. Although ISO/IEEE 11073 and ASTM F-2761 (Integrated Clinical Environment, ICE) are two applicable standards, the former has not been widely adopted and the latter is still relatively new (2009). Many groups are tackling the problem of interoperability on their own by developing the hardware and software interfaces that enable device connectivity. Connecting with analog data ports requires appropriate hardware interfaces, analog-to-digital (A/D) converters, and filters to eliminate aliasing due to a mismatch between sampling rate and the frequency content of the signal being acquired. It also requires that the data be properly scaled to the voltage range of the A/D converter (microvolts to millivolts) to maximize the resolution. Digital data is available from some devices through connection to serial (RS-232 or USB) or Ethernet (802.3) ports, or using wireless (e.g., 802.11b/g or Bluetooth) communications. Although these approaches provide the opportunity to individually interface with a variety of devices in the ICU, a system that provides comprehensive, cross-manufacturer medical device integration for the care of a single critically ill patient at the bedside is not available.

Several third party systems have recently emerged specifically to help facilitate this comprehensive data acquisition and integration. For example, Bedmaster XA (Excel Medical Electronics, Jupiter, and Florida) is a product that can be used to collect medical device data with access through the hospital local area network. First introduced in 1999 to assist clinicians by automatically acquiring a patient's vital signs from a GE/Marquette patient monitor, the current system works with both GE and Philips patient monitors acquiring parameter data (such as vital signs) from every five seconds to every hour. DataCaptor (Capsule Technologie, Paris, France) is another similar product that can be used to collect medical device data.

Time synchronization of the data is a critical feature for multimodal data acquisition from different devices and monitors. Without a “master clock” ensuring that all the values and waveforms acquired at the same time “line up” exactly in synch, interpreting the information and understanding the interrelationships are difficult, if not impossible. There are two issues. First, when data is being acquired from different devices, each with its own internal clock, the time stamps of data acquired simultaneously can all be different. Time synchronization is therefore necessary when simultaneous analog and digital data streams are acquired in order to align the data. Second, even when acquiring data from a single patient monitor, time drifting from natural degradation, daylight savings time, or incorrect adjustments made by the clinical staff need to be corrected. The Unity Time feature in the Bedmaster XA system manages time synchronization by insuring the accuracy of the time clocks on all GEMS devices connected to the Unity Network. Unity Time functions in conjunction with a NTP (Network Time Protocol) server, as specified by the medical facility. Time clocks on all GEMS patient monitors connected to the unity network are automatically reset to the NTP server at a time interval selected by the hospital. It is primarily these obstacles that have limited wide spread adoption of multimodal monitoring technology in the ICU.

## 6. Data Acquisition and Integration Systems for Research

Commercial off-the-shelf products do not support high-resolution physiologic data acquisition, archiving, or annotation with bedside observations for clinical applications. Such systems have been developed in academic settings though mainly for clinical research. Because they are not open source, most of these systems are not readily available. This has resulted in considerable duplication of effort in software development for acquiring and archiving physiological data.

There have been a variety of efforts ranging from developing and testing of new mathematical and analytical tools, to hardware/software solutions for patient data acquisition, archiving, and visualization. A complete listing is beyond the scope of this review but several stand out (see Table 2). For example, Tsui and colleagues developed a system for acquiring, modeling, and predicting ICP in the ICU using wavelet analysis for feature extraction and recurrent neural networks to compute dynamic nonlinear models [31]. Smielewski and colleagues from Cambridge University have developed the Intensive Care Monitoring (ICM+) system; configurable software based on MATLAB (The Mathworks, Natlick, MA) that allows real-time acquisition, archiving, and analysis of multimodal data that can then be displayed in several ways including simple trends, cross histograms, correlations, and spectral analysis charts. The software is intended for research so it stores the raw signals acquired from bedside monitors for subsequent reprocessing, thus providing the means of building a data repository for testing novel analytical methods [32].

Others have focused on multimodal data collection linked with clinical annotation. In London, Gade and his colleagues reported the development of the Improved Monitoring for Brain Dysfunction in Intensive Care and Surgery (IBIS) data library that contained continuous EEG signals, multimodal evoked potential recordings, and ECG [33]. The system captured trend data from patient monitors, laboratory data, and some clinical annotations. In 2001, Kropyvnytskyy and colleagues [34] reported a similar system in Boston called SWE for sampling, data display (WAVE), and ECG processing. SWE was initially developed for the MIT-BIH arrhythmia database (and now used for publicly available databases on the National Institutes of Health-sponsored* PhysioNet* website).

Sorani and colleagues from San Francisco General Hospital [35] also developed a system that captured over 20 physiological variables (plus date, time, and annotated clinical information) from Viridia bedside monitors (Philips), Licox tissue oxygen monitors (Integra NeuroSciences, Plainsboro, NJ), and Draeger ventilators (Luebeck, Germany). Data was collected automatically at 1-minute intervals and was output into text files. Monitoring data was integrated by special custom developed middleware (Aristein Bioinformatics). Goldstein and colleagues from Oregon Health Sciences University developed a physiologic data acquisition system that could capture and archive parametric data (such as blood pressure and heart rate) along with the underlying waveforms to assess dynamic changes in the patient's physiologic state [36]. The system consisted of a laptop computer, a standard PCMCIA serial card (Socket Communications, Newark, CA), RS232 serial interface cables, and custom software. The system acquired analog data from devices incorporating antialiasing filters along with analog-to-digital conversion. Parametric data was sampled at a rate of 0.98 Hz and continuous wave data either at 500 Hz (ECG) or 125 Hz (pressures, arterial saturation, and respiration). Software managed communications with the monitoring devices; the collected signal data were sent to a patient data server and workstation where the files were archived. Standard analytical software packages, such as MATLAB, facilitated advanced mathematical analyses including time and frequency domain methods and linear and nonlinear signal metrics. Annotation of important clinical events, such as changes in a patient's condition or timing of drug administration, was limited. In 2006, the same group reported the next generation of their system that added event markers and clinical annotation [37].

Moody and Mark from Massachusetts General Hospital initially reported on their initial efforts in developing the MIMIC (Multiparameter Intelligent Monitoring for Intensive Care) database [38]. An updated version, MIMIC II, was reported in 2002 [39]. Each record consisted of four continuously monitored waveforms (two leads of ECG, Arterial Blood Pressure, and Pulmonary Artery Pressure) sampled at 125 Hz, along with other basic parameters (heart rate, oxygen saturation, and cardiac output) collected every minute. The waveforms and parameters were originally acquired from Philips bedside patient monitors using their RDE software tool. Using a customized archiving agent, the waveform and parameter data were later stored onto storage drives and converted from Philips' proprietary RDE data format into an open-source format (WFDB), thereby making it accessible to others for research. Various tools were used to analyze data. The 1-minute parameter data were processed using wavelet analysis to identify potentially relevant clinical events. Matching waveform records to clinical data was based on unique identifiers such as medical record numbers, dates of admission, and patient names. A text search engine was created to allow users to query the database for key words and patterns of interest. In 2011, this MIMIC II database was made public and is available for research [40].

While MIMIC II represents a major achievement, because physiological data and clinical annotations are collected separately, the two datasets are poorly synchronized. Also, physiological data and clinical annotations have different time “granularity,” making it difficult to retrospectively determine the timing of a clinical event down to seconds. Reconstructing the clinical context with these limitations results in correlations being largely speculative. In parallel to MIMIC II, Meyer and Colleagues at MGH, as part of the “OR of the Future” project, introduced a system for the operating room to perform integration and display of data from a variety of disparate sources, including hospital information systems, patient monitors, surgical equipment, and a location tracking system [41]. At the core of this system is custom integration software (LiveData OR RTI Server, Live-Data, Inc., Cambridge, MA, USA) that is used to capture in real-time all device data (except from infusion pumps), including detailed physiological waveform data and all data elements, without data loss. Custom interfaces are needed for devices with proprietary data formats. Data are maintained in a relational database with an archive of all captured OR data, including trends and full resolution waveforms, information from the location tracking system, and patient and scheduling information (from multiple hospital information systems). This automated database allows time-based playback and analysis of the events of the surgery. Selected data are displayed in real-time on an integrated display created using scalable vector graphics.

In 2011, Feng and colleagues developed the intelligent System for Neuro-Critical-Care (iSyNCC) to facilitate multimodal data acquisition and transmission across a local network for storage in a database and computational analysis. The system includes an “artifact removal” module to ensure high quality data and the ability to provide short term data forecasting (for example of ICP elevations). There is also a “Recovery Outcome Prediction” module to estimate patient long-term outcomes based on integration of the clinical records and physiological data [42].

## 7. Developing the Integrated Medical Environment

**Figure 1:**
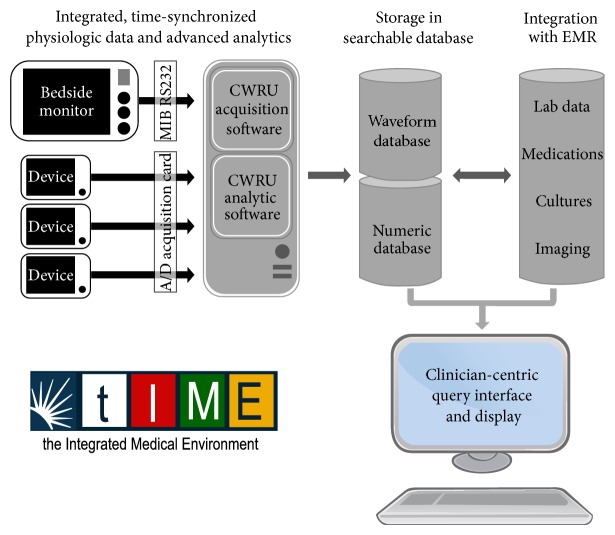
Schematic illustration of the integrated medical environment (tIME).

At University Hospitals Case Medical Center and Case Western Reserve University, we have focused on overcoming many of the obstacles described and putting everything together: high-resolution physiologic data acquisition, integration, processing, archiving, annotation with bedside observations for clinical applications, and visualization. The Integrated Medical Environment™ (tIME™) (Figure 1), is a new open source architecture that we believe can provide the backbone for the ICU of the future. Specifically tIME™ provides (1) real-time data acquisition, integration, time-synchronization, and data annotation of multimodal physiological waveform data (both analog and digital) from a variety of medical devices and bedside monitors using custom developed parsing algorithms. Both the waveform data and the extracted parametric numeric data are displayed using real-time algorithms developed by our group and simultaneously stored in a local database for easy access, retrieval, and queries. The local database can connect and import into the hospital EMR using a secure HL7 data transfer protocol; (2) a new critical care open middleware informatics architecture that facilitates complex systems analysis methods and data mining capabilities for hypothesis generation and testing; and (3) a clinician-centric visual display and interface, to present an integrated overview of the patient state (past, present, and predicted futures) so that providers can make sensible decisions at the bedside based on all the data. Only when all of these components work in concert will we be able to fully harness the power of information technology to improve patient outcomes in the ICU.

## 8. Conclusions

While there have been major improvements in intensive care monitoring, including the development of enterprise Clinical Information Systems, the medical industry, for the most part, has not incorporated many of the advances in computer science, biomedical engineering, signal processing, and mathematics that many other industries have readily embraced. Acquiring, synchronizing, integrating, and analyzing patient data remains frustratingly difficult because of insufficient computational power and a lack of specialized software, incompatibility between monitoring equipment, and limited data storage. All of these technical problems are now surmountable. Today, we are on the verge of the data-intensive science era in which hypotheses will be generated automatically among the enormous amount of data available by using computational science with inductive reasoning. In this new era, information technology enabling the development of an integrated critical care informatics architecture that supports clinical decision-making at the bedside will be essential.

## Figures and Tables

**Table 1:** Timeline of computers in the ICU.

	Computer	
1946	ENIAC introduced	
1951	IBM ERA 1103 introduced	
1966	HP 2115 introduced	

	Electronic medical record	Computer systems for the ICU

1961		Clinical decision support for diagnosis of hematologic disorders (Lipkin)
1964		Marquette Electronics founded
1965		Computerized respiratory monitoring (Stacy and Peters)
1966		Computerized collection of vital signs (Shubin)
1969	IDX System founded for revenue cycle management (utilizing MUMPS)	
1973		Computer assisted monitoring with trend analysis (Lauwers)
1975		HP introduces Patient Data Management System (PDMS)
1976		Clinical decision support for management of critically ill surgical patients (Siegel)
1977	The Medical Record (TMR), Duke University Regenstrief Medical Record System (RMRS), Indiana University	
1978	Problem Oriented Medical Record (POMR), University of Vermont Computer Stored Ambulatory Record (COSTAR), Harvard University (MUMPS)	
1979	Epic Systems founded (MUMPS)	
1980		Computerized management system for ICU (Manzano)
1983	Health Evaluation through Logical Processing (HELP), University of Utah	
1986		Computerized system for automating blood transfusion (Sheppard)
1997	Veterans Health Information Systems and Technology Architecture (VistA) founded	HP introduces Carevue system Capsule Technologie introduces DataCaptor
1999		Excel Medical Electronics introduces Bedmaster XA
2000	MedicaLogic founded	

	Clinical Information Systems	

2003	GE's Centricity Critical Care system introduced	
2007	Philips IntelliVue Clinical Information Portfolio (ICIP) Critical Care introduced	

**Table 2:** Data acquisition and integration systems for research.

1996	Moody and colleagues [38]	Multiparameter Intelligent Monitoring for Intensive Care (MIMIC)
1998	Tsui and colleagues [31]	Acquisition, modeling, and predicting ICP
2000	Gade and colleagues [33]	Improved Monitoring for Brain Dysfunction in Intensive Care and Surgery (IBIS)
2001	Kropyvnytskyy and colleagues [34]	Sampling, data display (WAVE), and ECG processing (SWE) system
2003	Goldstein and colleagues [36]	Data acquisition system to capture both parametric data and underlying waveforms
2007	Sorani and colleagues [35]	Aristein Bioinformatics system
2007	Meyer and colleagues [41]	“OR of the Future”
2008	Smielewski and colleagues [32]	Intensive Care Monitoring (ICM+) system
2011	Feng and colleagues [42]	intelligent System for Neuro-Critical-Care (iSyNCC)
